# Cerebrospinal fluid CXCL13 as a diagnostic marker of neuroborreliosis in children: a retrospective case-control study

**DOI:** 10.1186/s12974-017-0948-9

**Published:** 2017-08-31

**Authors:** M. M. Remy, N. Schöbi, L. Kottanattu, S. Pfister, A. Duppenthaler, F. Suter-Riniker

**Affiliations:** 1 Institute of Infectious Diseases, University of Bern, Friedbühlstrasse 51, 3001 Bern, Switzerland; 2 Department of Pediatrics, Bern University Hospital, University of Bern, Bern, Switzerland

**Keywords:** CXCL13, Lyme neuroborreliosis, Cerebrospinal fluid, Antibody index, Sensitivity, Specificity

## Abstract

**Background:**

Lyme neuroborreliosis (LNB) is a frequent manifestation of Lyme disease in children and its current diagnosis has limitations. The elevation of the chemokine CXCL13 in the cerebrospinal fluid (CSF) of adult patients with LNB has been demonstrated and suggested as a new diagnostic marker. Our aim was to evaluate this marker in the CSF of children with suspected LNB and to determine a CXCL13 cut-off concentration that would discriminate between LNB and other central nervous system (CNS) infections.

**Methods:**

For this single-center retrospective case-control study we used a diagnostic-approved ELISA to measure CXCL13 concentrations in the CSF of 185 children with LNB suspicion at presentation. Patients were classified into definite LNB (cases), non-LNB (controls with other CNS affections), and possible LNB. A receiver-operating characteristic curve was generated by comparison of cases and controls.

**Results:**

CXCL13 was significantly elevated in the CSF of 53 children with definite LNB (median 774.7 pg/ml) compared to 91 control patients (median 4.5 pg/ml, *p* < 0.001). A cut-off of 55 pg/ml resulted in a sensitivity of 96.7% and a specificity of 98.1% for the diagnosis of definite LNB and the test exhibited a diagnostic odds ratio of 1525.3. Elevated CSF CXCL13 levels were also detected in three controls with viral meningitis (enterovirus *n* = 1, varicella-zoster virus *n* = 2) while other CNS affections such as idiopathic facial palsy did not lead to CXCL13 elevation. Of the 41 patients with possible LNB, 27% had CXCL13 values above the cut-off of 55 pg/ml (median 16.7 pg/ml).

**Conclusions:**

CSF CXCL13 is highly elevated in children during early LNB as previously shown in adults. CXCL13 is a highly sensitive and specific marker that helps to differentiate LNB from other CNS affections in children.

## Background

Lyme disease is the most common vector-borne disease in the Northern Hemisphere [1, 2]. It is caused by *Borrelia burgdorferi* sensu *lato* spirochetes [3], which are transmitted to humans by ticks. Early Lyme neuroborreliosis (LNB) is a frequent manifestation of Lyme disease in children [4]. It presents a few weeks to months (mostly 4–6 weeks) after a tick bite with neurological deficit, i.e., aseptic meningitis, radiculitis, or loss of function of cranial nerves [5]. The most common neurological manifestations of LNB in European children are acute facial nerve palsy (55–61%), other cranial nerve palsies, and lymphocytic meningitis (27%) [4–8]. Signs and symptoms of early LNB typically last for less than 6 months, and the natural course of the disease is often self-limiting [9].

Laboratory confirmation of LNB is hampered by the low yield of bacterial culture and the poor sensitivity of polymerase chain reaction (PCR) in the cerebrospinal fluid (CSF) [10, 11]. According to the case definition of the European Federation of Neurological Societies (EFNS) [12], intrathecal production of antibodies against *Borrelia burgdorferi* (B.b.) must be proven to confirm the diagnosis of definite LNB. Therefore determination of Borrelia-specific antibody index (AI) in the CSF has become the traditional diagnostic gold standard, despite limitations such as low sensitivity in the very early phase of the disease [5, 13, 14] and persistence for years after eradication of the infection [15, 16]. In addition, it was shown that up to 20% of patients with proven *Borrelia burgdorferi* infection have negative AI [17]. In such cases when suspicion of neuroborreliosis persists, a seroconversion in serum has to be demonstrated after 6 weeks to confirm the diagnosis of LNB [12], causing uncertainties for patients and delaying treatment. Furthermore, early antibiotic therapy can also affect the humoral response and IgM-IgG switch resulting in negative-specific AI and negative serum IgG [17]. Additional biomarkers with high sensitivity and specificity are therefore necessary to improve diagnostic procedures.

Recently, measurement of the chemokine CXCL13 in CSF samples of suspected LNB patients has been identified as a new potential diagnostic tool [18–28]. CXCL13 is a B-cell chemoattractant expressed in secondary lymphoid organs including the central nervous system (CNS) [29]. It is synthesized in early LNB in considerable amounts into the CSF alongside less clinically relevant chemokines and cytokines [28, 30–32]. CXCL13 promotes the secretion of cytokines that facilitate humoral reactions and migration of B lymphocytes and plasma cells into the CSF [18]. During LNB, B-cells reaches up to 80% of total cells in CSF, clearly exceeding other CNS infections and inflammatory diseases [29, 33, 34]. Accordingly, a strong up-regulation of CXCL13 was observed in the CSF of early LNB patients and not for other etiologies of CNS inflammation [20, 23]. In contrast, no correlation between blood levels of CXCL13 and early LNB was demonstrated [35]. CSF CXCL13 elevation could even be detected before the intrathecal production of B.b.-specific antibodies, confirming the key role of this chemokine in B-cell migration to the infected CNS [22, 26]. By comparing CXCL13 levels in the CSF of patients with early LNB or with other CNS inflammatory diseases, several studies have determined CXCL13 cut-off levels for the diagnosis of early LNB. These cut-offs vary between 61 pg/ml and 1229 pg/ml [20, 21, 23, 25, 27] and were mostly determined for adult patients with inclusion of few pediatric patients [21]. More studies are therefore required to find a universal cut-off and to firmly establish the diagnostic value of CSF CXCL13 in LNB, especially in children.

The objective of our study was to evaluate the clinical relevance of measuring CXCL13 in the CSF of children with suspected LNB in the endemic region of Bern, Switzerland, and to establish a cut-off value for the diagnosis of early LNB in children. Using specific antibody index as a gold standard, we compared concentrations of CXCL13 among CSF samples of patients with definite LNB, possible LNB, and non-LNB controls.

## Methods

### Setting and patients

This is a single-center retrospective case-control study in the endemic region of Bern, Switzerland, over the period of January 2012 to December 2016. Children (0 to 18 years of age) with suspicion of neuroborreliosis and for whom frozen CSF samples were available, were retrospectively identified using the electronic database of the Institute for Infectious Diseases of the University of Bern, Switzerland. Clinical data were obtained from the archived charts or the electronic database of the Children’s University Hospital Bern, Switzerland. Patients were divided into the following subgroups according to the European Federation of Neurological Societies (EFNS) guidelines [12]: definite LNB (cases), non-LNB (controls), and possible LNB.

Definite LNB was diagnosed using the following criteria: (i) patients had symptoms consistent with LNB and other relevant differential diagnoses were excluded, (ii) presence of mononuclear pleocytosis (total cell count >5/μl), and (iii) intrathecal production of Borrelia-specific antibodies. Possible LNB was diagnosed if only two out of three criteria were fulfilled. When less than two criteria or when a relevant differential diagnosis was present, LNB was excluded and the case was moved to the control group (non-LNB). Relevant differential diagnoses were defined as: spondylodiscitis, Guillain-Barré syndrome, mastoiditis, hereditary neuropathy, muscular atrophy, stroke, esophoria, erythromelalgia, infections with enterovirus, tick-borne encephalitis virus (TBEV), or varicella-zoster virus (VZV). Patients with malignancies or with CNS symptoms longer than 6 months were excluded from the study. LNB symptoms were defined by one or several of the following symptoms: facial palsy, headache, cranial nerve palsy, meningism, radiculitis, myelitis, ataxia, hemiparesis, fever, blurred vision, and cauda equina syndrome. Known antibiotic pre-treatment was not considered an exclusion criteria since patients who received antibiotics prior to lumbar puncture were not exhaustively identified in our data bank.

### CSF and serum laboratory analyses

Routine CSF and serum laboratory analyses were performed at the University of Bern, Switzerland, respectively, in the Center for Laboratory Medicine (cell count, protein) and in the Institute of Infectious Diseases (serology and antibody index). Serum and CSF samples were subsequently stored at −20 °C at the Institute of Infectious Diseases. A screening test for Borrelia was first performed on fresh serum samples using enzyme-linked immunosorbent assays (ELISA) for IgG and IgM (recomWell, Mikrogen, or Virion/Serion) according to the manufacturer’s instructions. In case of positive screening test or persistent suspicion of early neuroborreliosis, CSF/serum antibody index was determined. In this case, the presence of Borrelia-specific antibodies in simultaneously sampled serum and CSF probes was determined using IgG and IgM ELISA according to the manufacturer’s instructions. Two different ELISA kits were used over the period of the present study (IDEIA, OXOID until May 2014, and Virion/Serion after June 2013). The antibody index (AI) was considered positive when ≥0.3 (IDEIA, OXOID) or when ≥1.5 (Virion/Serion).

CSF CXCL13 concentrations were measured by the first diagnostic-approved CXCL13 ELISA (EUROIMMUN AG, Luebeck, Germany) according to the manufacturer’s instructions. CSF samples which had been stored for maximum 3 years at −20 °C were allowed to thaw at room temperature and measurements were performed neat in duplicates (intra-assay variation coefficients <15%). In a few cases, a dilution 1:5 was needed to analyze the samples due to low available volume; this had no influence on the test result. Incubations were done at room temperature. CXCL13 values below the detection limit of 4.6 pg/ml were assigned a value of 4.5 pg/ml. When optical density (OD) values exceeded the OD value of the highest standard, samples were further diluted 1:10 and 1:100 to obtain accurate concentrations of CXCL13. Results were obtained within 4 h.

### Statistics

Statistical analyses and ROC curves were performed using GraphPad Prism 6.0 (GraphPad Software, San Diego, USA). Diagnostic test evaluation was performed using MedCalc online calculator (MedCalc Software, Ostend, Belgium). Data are presented as a median (range) value or a number (%) value. Comparison of means was performed using non-parametric Mann-Whitney test. *P* values <0.05 were considered significant.

## Results

### Patients

Initially, 207 children with suspicion of Lyme neuroborreliosis at presentation were retrospectively identified for the present study, of which 22 had to be excluded due to malignancy or prolonged symptom duration. The remaining 185 patients were retained, and their CSF was analyzed for CXCL13. Details on sex, age, clinical presentation, and duration of symptoms until diagnostic lumbar puncture are presented in Table 1. After routine analyses, 53 patients (29%) were diagnosed with “definite LNB,” i.e. positive antibody index (AI), CSF pleocytosis (CSF cell count >5 cells/μl), and neurological symptoms [12] while 91 patients (49%) were classified as controls in a “non-LNB” group (negative AI and no pleocytosis or presence of a differential diagnosis). Some of these control patients had viral meningitis or encephalitis due to varicella-zoster virus (VZV, *n* = 4), enterovirus (*n* = 20), or tick-borne encephalitis virus (TBEV, *n* = 5), while others had inflammatory diseases (*n* = 8), idiopathic facial palsy (*n* = 19), primary headache (*n* = 11), or other diseases (*n* = 29). Inflammatory diseases included three Guillain-Barré Syndrome, three retrobulbar optic neuritis, one acute disseminated encephalomyelitis (ADEM), and one parainfectious brainstem encephalitis.

**Table 1:** Baseline characteristics of the patient groups

	AI	Pleo.	n (M/F)		Age^a^ (years)		CSF cell count^a^ (cells/ul)		Mononuclear cells^a^ (%)		Symptoms duration^a^ (days)		Facial palsy^b^	Headache^b^	Cranial nerv palsy^b^	Negative serology^b^
Definite LNB	+	+	53 (30/23)		7.7 (2.2–15.5)		154.0 (8–1289)		98.5 (74–100)		9.0 (0–109)		35 (66)	15 (28)	1 (2)	5 (9)
Possible LNB			41 (17/24)		8.3 (1.8–15.7)		47.0 (3–999)		96.4 (10–100)		4.0 (0–128)		8 (20)	20 (49)	4 (10)	25 (61)
AI neg.	−	+	27 (10/17)		8.8 (1.8–15.7)		76.0 (7–999)		97.7 (10–100)		3.0 (0–14)		5 (19)	13 (48)	4 (15)	13 (48)
AI nd.	nd	+	13 (6/7)		6.2 (2.7–15.5)		31.0 (16–448)		96.0 (59–100)		6.0 (1–128)		2 (15)	7 (54)	0 (0)	12 (92)
AI pos.	+	−	1 (1/0)		7.9		3.0		nd		14.0		1 (100)	0 (0)	0 (0)	0 (0)
Non-LNB			91 (49/42)		10.0 (1.1–17.7)		2.0 (0–1174)		75.7 (18–99)		3.0 (0–166)		23 (25)	33 (36)	6 (7)	69 (76)
VZV	−	−/+	4 (3/1)		7.8 (1.8–17.7)		7.5 (0–579)		95.9 (92–99)		5.5 (0–10)		2 (50)	1 (25)	0 (0)	4 (100)
Enterovirus	−	−/+	20 (10/10)		7.2 (1.1–15.2)		194.0 (0–1174)		76.5 (18–98)		1.0 (0–21)		0 (0)	13 (65)	0 (0)	17 (85)
TBEV	−	+	5 (4/1)		10.8 (5.6–15)		80.0 (43–104)		71.6 (28–90)		3.0 (2–5)		0 (0)	5 (100)	0 (0)	3 (60)
Inflammatory	−	−	8 (1/7)		10.4 (5–15.5)		2.0 (0–5)		nd		2.5 (1–31)		0 (0)	0 (0)	0 (0)	5 (63)
Facial palsy	−	−	19 (11/8)		11.7 (1.5–15.7)		2.0 (0–5)		nd		3.0 (0–38)		19 (100)	0 (0)	0 (0)	14 (74)
Headache	−	−	11 (3/8)		13.8 (6.1–16.9)		0.0 (0–3)		nd		31.0 (0–166)		0 (0)	11 (100)	0 (0)	7 (64)
Others	−	−	24 (17/7)		6.6 (2.1–17.4)		1.0 (0–5)		nd		4.0 (0–162)		2 (8)	3 (13)	6 (25)	19 (79)
Total			185 (96/89)		8.2 (1.1–17.7)		34.0 (0–1289)		97.1 (10–100)		5.0 (0–166)		66 (36)	68 (37)	11 (6)	99 (54)

Symptoms duration is until the diagnostic lumbar puncture

Negative serology means negative Borrelien-specific IgM and IgG screening test in blood at initial work-up

The “Inflammatory” group included three Guillain-Barré syndrome, three retrobulbar optic neuritis, one acute disseminated encephalomyelitis (ADEM), and one parainfectious brainstem encephalitis

*LNB* Lyme neuroborreliosis, *AI* antibody index, *Pleo.* pleocytosis (>5 cells/μl in CSF), *n* numbers, *M/F* male/female, *neg.* negative, *nd* not determined, *pos.* positive, *VZV* varicella-zoster virus, *TBEV* tick-borne encephalitis virus

^a^Data are presented as median (range)

^b^Data are presented as number (%)

Forty-one additional patients (22%) were classified as “possible LNB” as they fulfilled only two out of three criteria for LNB [12]. This included 27 patients with pleocytosis and a negative AI, 13 patients with pleocytosis and no AI (not performed due to negative serology or lack of material), and 1 patient with a positive AI and no pleocytosis. In these cases, no final diagnosis could be established.

### Diagnostic evaluation of CSF CXCL13 in patients with definite LNB versus non-LNB

**Fig. 1:**
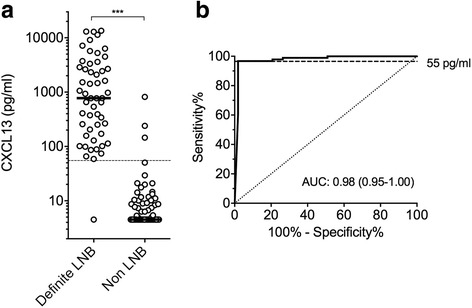
Diagnostic value of CSF CXCL13 in patients with definite LNB versus non-LNB. **a** CXCL13 concentrations in the CSF of pediatric patients with definite LNB versus control non-LNB patients. Black lines indicate medians. Values lower than the detection limit were arbitrarily assigned a value of 4.5 pg/ml. The dashed line indicates the optimal cut-off at 55 pg/ml. *LNB* Lyme neuroborreliosis. Means were compared by a Mann-Whitney test. ***P < 0.001. **b** Receiver-operating characteristic (ROC) curve of CSF CXCL13 levels from definite LNB patients versus control non-LNB patients. The dashed line indicates the optimal cut-off at 55 pg/ml. *AUC* area under the curve (95% confidence interval)

The median CXCL13 concentration in the CSF of patients with definite LNB was 774.7 pg/ml and values ranged from 58.9 to 13487.0 pg/ml with 1 outlier at 4.5 pg/ml (Table 2 and Fig. 1a). In comparison, non-LNB patients including patients with other confirmed CNS diseases had a median CSF CXCL13 concentration of 4.5 pg/ml (range 4.5–816 pg/ml). Means were highly significantly different (*p* < 0.001, Fig. 1a).

**Table 2:** CXCL13 concentrations in different disease groups

	AI	Pleo.	n	CXCL13^a^ (pg/ml)		CXCL13>CO^b^
Definite LNB	+	+	53	774.7 (4.5–13487)		52 (98)
Possible LNB			41	16.7 (4.5–1418.6)		11 (27)
AI neg.	−	+	27	25.0 (4.5–384.1)		9 (33)
AI nd.	nd	+	13	8.1 (4.5–1418.6)		2 (15)
AI pos.	+	−	1	13.9		0 (0)
Non-LNB			91	4.5 (4.5–816.1)		3 (3)
VZV	−	−/+	4	78.4 (4.5–239.9)		2 (50)
Enterovirus	−	−/+	20	5.8 (4.5–816.1)		1 (5)
TBEV	−	+	5	8.7 (6.7–19.9)		0 (0)
Inflammatory	−	−	8	4.5 (4.5–21.1)		0 (0)
Facial palsy	−	−	19	4.5 (4.5–14.6)		0 (0)
Headache	−	−	11	4.5 (4.5–7.4)		0 (0)
Others	−	−	24	4.5 (4.5–50.4)		0 (0)
Total			185	10.4 (4.5–13487)		66 (36)

The “Inflammatory” group included three Guillain-Barré syndrome, three retrobulbar optic neuritis, one acute disseminated encephalomyelitis (ADEM), and one parainfectious brainstem encephalitis

*CO* cut-off determined at 55 pg/ml in our study, *LNB* Lyme neuroborreliosis, *AI* antibody index, *Pleo.* pleocytosis (>5 cells/ul in CSF), *n* numbers, *neg.* negative, *nd* not determined, *pos.* positive, *VZV* varicella-zoster virus, *TBEV* tick-borne encephalitis virus

^a^Data are presented as median (range)

^b^Data are presented as number (%)

To assess the diagnostic performance of CXCL13, a receiver-operating characteristic (ROC) curve was drawn for cases (definite LNB) versus controls (non-LNB, Fig. 1b). The optimal cut-off for the diagnosis of definite LNB was 55 pg/ml with a sensitivity of 96.7% and a specificity of 98.1% (Table 3). The positive and negative likelihood ratios [36, 37] of the CSF CXCL13 test were, respectively, 29.8 and 0.02 resulting in a high diagnostic odds ratio of 1525.3 [38].

**Table 3:** Accuracy of CSF CXCL13 test to diagnose definite LNB in children using a 55 pg/ml cut-off

Sensitivity %	96.7 (90.7–99.3)
Specificity %	98.1 (89.9–100.0)
Positive LR	29.8 (9.8–90.6)
Negative LR	0.02 (0.0–0.14)
Diagnostic odds ratio	1525.3 (154.6–15047.4)

Definite LNB cases (*n* = 53) were compared to control non-LNB cases (*n* = 96)

*LR* likelihood ratio. 95% confidence interval is indicated in parentheses

### CSF CXCL13 in controls and possible LNB cases

**Fig. 2:**
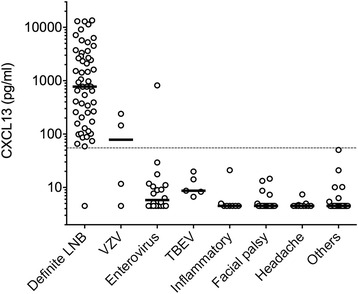
CSF CXCL13 concentrations in various control groups. Non-LNB patients were further classified in various disease groups according to the presence of a differential diagnosis. Definite LNB patients are shown for comparison. Black lines indicate medians. Values lower than the detection limit were arbitrarily assigned a value of 4.5 pg/ml. The dashed line indicates the optimal cut-off at 55 pg/ml. The “Inflammatory” group included three Guillain-Barré syndrome, three retrobulbar optic neuritis, one acute disseminated encephalomyelitis (ADEM), and one parainfectious brainstem encephalitis. *VZV* varicella-zoster virus, *TBEV* tick-borne encephalitis virus

Within the 91 patients of the “non-LNB” group, only 3 patients (3%) had a CSF CXCL13 concentration above the 55 pg/ml cut-off (Table 2, Fig. 2). Two of these patients were diagnosed with varicella by PCR amplification in the CSF and their CXCL13 levels were 239.9 and 145.1 pg/ml. In contrast the other two patients with VZV had very low CXCL13 concentrations (11.7 and 4.5 pg/ml). One patient with enterovirus infection also had a highly elevated level of CXCL13 (816.1 pg/ml) while the other 19 cases of enteroviral infection showed low CSF CXCL13 concentrations with a maximum of 29.3 pg/ml (median 5.8 pg/ml). Median CXCL13 concentrations in the CSF of patients with TBEV was 8.7 pg/ml while inflammatory diseases, facial palsy of unknown origin, primary headache, and other diseases had median CXCL13 levels of 4.5 pg/ml.

**Fig. 3:**
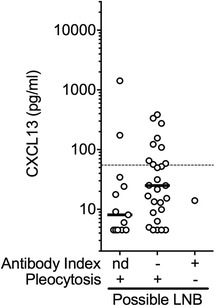
CSF CXCL13 concentrations in possible LNB patients. Patients with uncertain diagnosis of LNB according to the EFNS guidelines [12] and lacking a final differential diagnosis were regrouped in a possible LNB group which was subdivided according to antibody index and pleocytosis data. *nd* not determined. Black lines indicate medians. Values lower than the detection limit were arbitrarily assigned a value of 4.5 pg/ml. The dashed line indicates the optimal cut-off for the diagnosis of definite LNB at 55 pg/ml

In the possible LNB group, CXCL13 concentrations ranged from 4.5 to 1418.6 pg/ml (Table 2, Fig. 3). Patients were subdivided according to pleocytosis and AI availability. Most of them exhibited pleocytosis together with a negative AI (*n* = 27), of which nine (33%) had elevated CSF CXCL13 concentration. Another group of 13 possible LNB patients had pleocytosis but no determined antibody index due to a negative serology at initial work-up (in 92%, Table 1) and/or due to lack of material. Two (15%) of these 13 patients showed elevated levels of CXCL13 (Table 2, Fig. 3). The only patient with a positive index and no pleocytosis had a low CSF CXCL13 concentration (13.9 pg/ml). Possible LNB patients predominantly presented with headache along with meningitis (49%), acute facial palsy (20%), or other cranial nerve palsies (10%, Tables 1 and 4). None of these symptoms were associated with a clear CXCL13 pattern (Table 4). Results of CSF examination (Table 1) were highly variable but a lower percentage of mononuclear cells (<74%) was associated with a negative CSF CXCL13 result (Table 4). Also, patients presenting with symptoms with a duration of 5–7 days were for 73% positive for CXCL13 (Table 4). No other microorganisms could be found to confirm or infer LNB suspicion in these 41 patients with uncertain diagnosis.

**Table 4:** CXCL13 results in the possible LNB group according to various baseline characteristics

	*n* (%)	CXCL13 > CO
Possible LNB	41 (100)	11 (27)
Leading symptoms		
Facial palsy	8 (20)	2 (25)
Headache	20 (49)	7 (35)
Cranial nerve palsy	4 (10)	1 (25)
Mononuclear cells		
≥74%	32 (78)	11 (34)
<74%	8 (20)	0 (0)
Symptoms duration		
<5 days	22 (54)	2 (9)
5–7 days	11 (27)	8 (73)
>7 days	8 (20)	1 (13)

Data are presented as number (%). *CO* cut-off determined at 55 pg/ml in our study, *LNB* Lyme neuroborreliosis

## Discussion

We demonstrate that CXCL13 is highly elevated in the CSF of children with definite LNB and confirm previous findings in adult and mixed population [18–28]. By comparison with pediatric patients bearing similar CNS symptoms, we show that the human diagnostic-approved CXCL13 ELISA (Euroimmun) has a very high diagnostic performance (odds ratio 1525.3, [38]) for the diagnosis of early LNB with a cut-off at 55 pg/ml.

In the literature, cut-offs range from less than 100 to more than 1229 pg/ml and were determined using a similar but not diagnostic-approved CXCL13 ELISA (Quantikine, R&D). A cut-off at 250 pg/ml had been twice suggested [23, 26]. The studies, however, included mostly adult patients with very different symptoms, i.e., asymptomatic HIV, bacterial meningitis, or conditions that also result in a CSF CXCL13 increase, i.e., neurosyphilis or *Cryptococcus neoformans* infections [25]. This might have led to the difference in cut-off compared to our pediatric study. Recently, Pícha et al. [27] have strictly compared LNB adult patients to patients with aseptic non-borrelial CNS infections, and their lowest proposed cut-off was 29 pg/ml with a sensitivity of 90% and a specificity of only 72%. Contrary to our study, both definite and possible LNB cases were included in the cases numbers to determine this cut-off and elevated CXCL13 values in the non-LNB group were not discussed.

Within our definite LNB cases, only one patient had a CSF CXCL13 concentration lower than the defined cut-off of 55 pg/ml. She was a 3-year-old girl who was treated for an erythema migrans with amoxicillin 2 weeks before the onset of an acute facial palsy. She had a positive AI but a low CSF cell count of eight cells per microliter and her CXCL13 value was 4.5 pg/ml. It is known that CSF CXCL13 levels quickly decline after the initiation of an antibiotic treatment with ceftriaxone or doxycycline [25, 39, 40]; whether amoxicillin has a comparable effect is unknown and needs further evaluation. Similarly, the only “possible LNB” patient with a positive index and no pleocytosis had been treated with amoxicillin 11 days before lumbar puncture, which could explain the lack of pleocytosis as well as the low CSF CXCL13 concentration (13.9 pg/ml). In children, antibiotic treatments are frequently used in case of febrile infections and the likelihood for a coincidence of antibiotics pre-treatment with the onset of early LNB is high. In our retrospective study, however, these treatments were poorly documented, constituting a limitation to our results’ interpretation. CXCL13 values in our definite LNB patients might also be lower than in other studies where only untreated LNB patients were included. Long-term storage of CSF samples could in theory affect chemokine concentrations too. Previous studies have, however, reported good stability of CSF CXCL13 over repeated freeze-thaws and in CSF samples kept frozen for up to 5 years [23, 25].

Patients with possible LNB and a negative AI had a very short history of symptoms (median 3 days), compared to 9 days for patients with definite LNB. Such short symptoms durations could explain the lack of intrathecal antibody production [17, 20] and highlight the benefits of measuring CXCL13 in extremely early cases. In adults, CXCL13 could indeed be detected very early and sometimes even before pleocytosis occurred [22, 26].

Our study also shows for the first time the expected percentage of mononuclear cells in the CSF of children with definite LNB (median 98.5%, range 74–100%, Table 1). Concordantly, all possible LNB patients with less than 74% mononuclear cells had a negative CSF CXCL13 result (Table 4), hence virtually excluding true LNB according to the low negative likelihood ratio of the test.

Concerning controls, CSF CXCL13 concentrations were elevated in two cases of varicella-zoster virus and one case of enterovirus infection, both of which were detected in the patients’ CSF by PCR. Borrelia serology at presentation was negative for these three patients and for the enterovirus case it was still negative 6 weeks after the onset of symptoms, excluding a concomitant Borrelia infection. Such a sporadic elevation of CXCL13 in the CSF of patients with viral meningitis (VZV, enterovirus, TBEV, herpes simplex virus) had already been observed in previous studies [19, 20, 23, 25, 26] and probably reflects a rare transient induction of CXCL13 in the virally infected CNS. In our 5 pediatric cases of TBEV, however, CSF CXCL13 remained negative. In the literature, CSF CXCL13 elevation was also demonstrated for other diseases such as chronic infections (HIV, neurosyphilis) and autoimmune diseases (including multiple sclerosis or anti-NMDAR encephalitis) [20, 23, 25, 39–43], which are more prevalent in the adult population and were not represented in our study; as well as CNS lymphomas and some bacterial infections [19, 20, 44], which show a very different clinical picture and were not included in our study.

The strength of the CXCL13 diagnostic test (Table 3) was determined for confirmed definite LNB cases in comparison to non-LNB controls and therefore should help refine the diagnosis in uncertain cases of LNB suspicion. Within all possible LNB cases, 27% of patients exhibited elevated CSF CXCL13 concentrations, which corresponds to a high likelihood of true LNB (Table 3). According to the EFNS guidelines, a positive serology for Borrelia would however be needed 6 weeks later in order to confirm the diagnosis of neuroborreliosis in these uncertain possible LNB cases with a negative AI. This was not routinely performed in our retrospectively studied population and could only be confirmed in 1 out of 11 cases; a lack of data which clearly limits the interpretation of our results. Since no other microorganisms could be found in these patients early LNB therefore remained a highly likely but unconfirmed diagnosis. Such cases will be further examined in a prospective study.

CSF CXCL13 test would be especially helpful in an emergency setting when children present with acute facial palsy or headache and meningitis (Table 4). Acute facial palsy is the most common finding in European children with early LNB [4, 8]; however, its first differential diagnosis is idiopathic facial palsy as 40–75% of pediatric unilateral facial paralysis have unknown etiology [45, 46]. In our controls, we had 19 cases of idiopathic cases which all showed negative CSF CXCL13 (Fig. 2, Table 2), reinforcing the strength of the CXCL13 diagnostic test in uncertain cases of facial palsy. Regarding pediatric emergencies, CSF CXCL13 diagnostic test also requires less biologic material and has faster turn-around time compared to the AI. As little as 150 μl of CSF and 4 h are needed to complete the test compared to 800 μl and up to several days for the AI.

## Conclusions

Our study on children with LNB symptoms supports previous findings in adults showing a high diagnostic performance of CSF CXCL13 for definite LNB cases, especially in case of negative serology, negative AI, or impossibility to perform the AI. The excellent negative likelihood ratio of the test (0.02) combined with pre-test probabilities should enable rapid exclusion of LNB in case of negative CSF CXCL13 results and absence of previous antibiotic treatment, while the high positive likelihood ratio (29.8) will also give a strong diagnostic lead [36, 37]. Therefore, we recommend the systematic use of the CXCL13 diagnostic-approved ELISA in children to help clinicians in taking prompt decisions regarding treatment.

## Abbreviations

AI: Antibody index
CNS: Central nervous system
CSF: Cerebrospinal fluid
EFNS: European Federation of Neurological Societies
ELISA: Enzyme-linked immunosorbent assays
LNB: Lyme neuroborreliosis
OD: Optical density
PCR: Polymerase chain reaction
TBEV: Tick-borne encephalitis virus
VZV: Varicella-zoster virus
